# Content validation of observer-reported sickle cell pain diaries (SCPD-CS and SCPD-CN): results from interviews with caregivers

**DOI:** 10.1186/s12955-021-01888-5

**Published:** 2021-11-17

**Authors:** Michelle K. White, April M. Foster, Miranda Bailey, Denise D’Alessio, Avery Rizio, Patricia Stebbins, Danielle St. Pierre, Cory Saucier

**Affiliations:** 1 QualityMetric Incorporated, LLC (Formerly Known As Optum Patient Insights), 1301 Atwood Ave, Suite 216E, Johnston, RI USA; 2 Novartis Pharmaceutical Corporation, One Health Plaza, East Hanover, NJ USA; 3 Formerly of Optum Patient Insights, 1301 Atwood Ave, Suite 311N, Johnston, RI USA

**Keywords:** Content validity, Daily diary, Sickle cell disease, Caregivers, Qualitative

## Abstract

**Background:**

Patients with sickle cell disease (SCD) experience daily pain and acute episodes known as sickle cell pain crises (SCPCs). The Sickle Cell Pain Diary-Caregiver Report (SCPD-C) is an observer-reported diary for use by caregivers of children ages < 12 years with SCD. This study reports on the content validity of the SCPD-C.

**Methods:**

The SCPD-C was developed based on a literature review, measurement expert input, and a patient advisory board including clinicians. Three rounds of interviews (including both concept elicitation and cognitive debriefing methodologies) were conducted with caregivers of children with SCD aged < 12 to evaluate the content validity of the SCPD-C.

**Results:**

Across three rounds of interviews, caregivers confirmed concepts in the SCPD-C and described observed impacts that were important and were added. Overall, caregivers evaluated the SCPD-C as easy to understand, with some minor adaptations for clarity. Additionally, the diary was split into two versions based on the child’s age and school enrollment status (SCPD-CS for school-aged and SCPD-CN for non-school age children).

**Conclusions:**

Caregivers provided valuable input that led to important additions and changes to the measures. The SCPD-CS and SCPD-CN are appropriate and fit-for-purpose observer-reported outcome measures of SCPC-related pain frequency and severity, and impacts on health-related quality of life.

## Introduction

Sickle cell disease (SCD) is a genetic, progressive disease affecting approximately 100,000 children and adults in the United States (US) [1]. SCD particularly affects African Americans and Hispanic Americans in the US, as 1 out of every 365 African-Americans are born with SCD and 1 out of every 16,300 Hispanic Americans are born with SCD [1–3]. Starting in early childhood, clinical features of SCD include pain, fatigue, cognitive difficulty, jaundice, hearing loss, eye damage, nausea, insomnia, susceptibility to infections, priapism, and asthma [4–8]. Children and adolescents with SCD experience impacts on psychological well-being, including high pain burden, depression, perceived health-related and racial stigma [9–11], and reduced health-related quality of life (HRQoL) compared to peers [12].

Vaso-occlusive crises, often referred to by patients as sickle cell pain crises (SCPCs), are a hallmark complication of SCD and are associated with increased risk of morbidity and mortality [8]. SCPCs occur as a consequence of vaso-occlusion, which occurs when the lumen of one or more vessels in the microvasculature is occluded due to the formation of a multi-cellular complex. Recent research has demonstrated that overexpression of adhesion molecules such as p-selectin, on the surface of the endothelium, leukocytes and platelets in patients with SCD, plays a key role in adhering the cells together and to the surface of the endothelium [4, 13, 14]. SCPCs are characterized by acute, often debilitating pain, and have been reported as the most problematic complication of SCD in children and adults [5]. SCPCs can lead to life-threatening events including stroke and acute chest syndrome [4, 5, 15], have been shown to impact patients’ quality of life and ability to function [8], and are a primary cause of healthcare resource utilization (HCRU) [6–8, 16, 17].

While many studies have focused on evaluating rates of mortality, medical expenditures, or HCRU (i.e., hospitalizations and emergency department visits) among patients with SCD [18–20], fewer studies have focused on outcomes such as HRQoL and daily functioning to provide a more complete understanding of the patient experience [21–23]. As newborn screening for SCD in the US has become routine [24] and infant mortality rates have decreased [25], the goal of treatment has shifted toward improving the quality of patients’ lives [26] rather than focusing only on reducing mortality. In recognition of these new treatment goals, it is important to ensure that clinicians and researchers have measurement strategies that appropriately assess the types of outcomes that are most relevant to patients. Reliance on hospital records and HCRU alone may not be sufficient to capture the totality of the patient experience, particularly for pediatric patients whose pain is often managed at home [7]. As such, alternative measures that provide a more comprehensive evaluation of the daily experience of patients with SCD, and in particular children with SCD, should be considered.

Daily diaries represent one way to evaluate outcomes that may be especially difficult to capture through medical records, during clinic visits, or with less-frequently administered surveys. Because SCD-related pain can occur with or without an SCPC, vary day-to-day, and the onset, severity, and impacts of SCPCs are both unpredictable and variable, evaluation of such experiences may be best measured through daily diaries. Prior studies using a daily diary self-report format for children with SCD have included measures primarily focused on capturing how pain is managed at home and how pain impacts daily and physical activities [16, 27–31]. Despite the utility of self-report daily diaries, there are limitations when this methodology is applied to specific populations. In particular, guidelines suggest that children < 5 years old cannot provide reliable and valid self-report data [32]. While children ages 5–11 may be able to self-report, they may experience difficulty understanding the survey content, which can affect the reliability of data obtained through these measures [32]. Moreover, research has suggested that when caregivers enter daily diary data on behalf of their children, adherence for completing the daily diary is increased [16]. Given the utility of daily diaries in assessing pain in children with SCD, along with the constraints that exist in obtaining self-reported data from children under the age of 12, assessment of pain and other impacts of SCD in this specific population may be best accomplished through use of a daily observer-reported outcome (ObsRO) diary.

The Sickle Cell Pain Diary—Caregiver Report (SCPD-C) was developed as a daily ObsRO measure for caregivers of children with SCD who are under the age of 12. The objective of this study was to evaluate the content validity of the SCPD-C, through a series of qualitative interviews with caregivers of children with SCD.

## Methods

### Diary development

A draft version of the daily diary, titled SCPD-C v.01, was developed using multiple resources. First, key concepts were identified from the literature, using a structured review of pre-existing instruments that measure pain and HRQoL in an SCD population, and a published conceptual model of SCD impacts [33]. Additionally, consultation with measurement experts, feedback collected during a day-long patient advisory board meeting that included clinicians, and individual discussions with clinicians helped to identify additional concepts which were used by the research team to draft the items.

The SCPD-C v.01 was intended to measure pain severity, pain duration, fatigue, medication use in children with SCD to manage pain, and impact of pain on the child’s HRQoL during an SCPC, as observed by the caregiver. The diary included 11 items that pertained to caregivers’ observations during an SCPC and the subsequent impact of the SCPC on their child’s HRQoL in the past 24 h. Most items either used a 5-point Likert scale (e.g., “Not at All” to “Extremely”) or were Yes/No. One item asked the caregiver to provide the approximate number hours and minutes of the observed SCPC. If signs of an SCPC were not observed in the past 24 h, the caregiver would complete one item using a 5-point Likert scale about non-SCPC-related pain their child may have experienced.

### Participant sample

The study was approved by the New England Independent Review Board, and informed consent was obtained from all participants. Participants were invited to participate in the study through collaboration with a healthcare research recruitment agency. Individuals were eligible to participate in the study if they were a primary caregiver of a child < 12 years of age with SCD, observed their child experiencing at least one SCPC in the 12 months prior to screening, spoke English fluently, and were willing and able to participate in a 60–90 min interview.

### Interview procedure

**Fig. 1:**
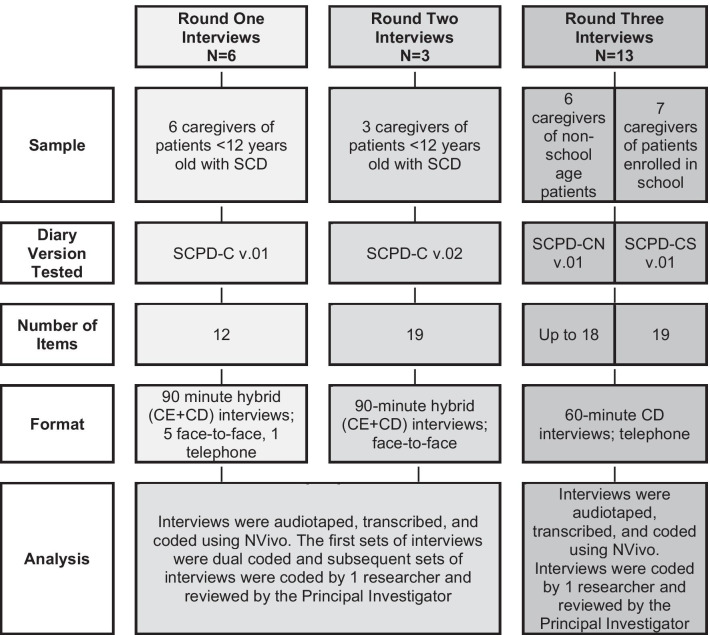
Description of study methodology, divided by interview round. *CD* cognitive debriefing, *CE* concept elicitation, *SCD* sickle cell disease, *SCPD-C* sickle cell pain diary-caregiver report, *SCPD-CN* sickle cell pain diary-caregiver report for non-school age children, *SCPD-CS* sickle cell pain diary-caregiver report for school age children

Interviews with caregivers of children with SCD were designed to collect data on the relevance, comprehensiveness, and comprehensibility of the SCPD-C. To meet this objective, two different interview approaches were employed: concept elicitation (CE) and cognitive debriefing (CD). An overview of each round of interviews is presented in Fig. 1. Interviews were conducted across three rounds in multiple US locations in-person and via telephone using semi-structured interview guides. All interviews were completed by experienced researchers with training in conducting qualitative interviews, and were audio-recorded with the permission of each participant. After the interviews were completed, caregivers received an honorarium in compensation for their time.

#### Concept elicitation

An open-ended CE approach was used across the first two rounds of interviews to explore concepts of SCD that were important to participants, helping to provide evidence that the SCPD-C is comprehensive in the concepts it includes and informing additional content. Caregivers were asked to report on their observations of their child’s daily experience of SCD symptoms and the impact of those symptoms on aspects of their child’s life. They were then asked to discuss their child’s symptoms, signs, or changes in behaviors during an SCPC, what a typical day looks like during and outside of an SCPC, and what treatment they seek when their child is experiencing an SCPC.

#### Cognitive debriefing

A CD approach was used in all three rounds of interviews to test the relevance and comprehensibility of each element of the diary (instructions, items, response options, and skip patterns). A think-aloud process was used: caregivers were asked to complete the diary, answering all items while verbalizing their thoughts about the item and its response options [34]. Caregivers were then asked to describe any aspects of the diary they found challenging or confusing; the interviewer also probed areas that appeared to be confusing based on caregivers’ verbal and non-verbal cues during the think-aloud process. Finally, the interviewer asked a set of structured queries to ensure the relevance and comprehensibility of any elements of the diary that had not already been discussed.

### Data analysis

Interview audio-recordings were transcribed verbatim, and transcripts were coded and analyzed using identical methodology for each round of interviews. All interview data were coded using NVivo version 11.0 software.

For the CE approach, interview data were content coded and analyzed using content thematic analysis [35]. This strategy is in accordance with the principles of grounded theory [36]. Saturation—the point at which no new relevant information emerges– was evaluated using a constant comparative approach, whereby initial interviews were analyzed and compared contemporaneously with subsequent interviews [36–38].

For the CD approach, after each interview, a Microsoft Excel spreadsheet was populated with any issues that emerged that suggested a change be made. Such issues included survey elements (item, response choice, etc.) perceived as confusing or difficult to answer, or suggestions to improve clarity. Each unique suggestion was recorded in a single row, with a separate column for each interview. Next, transcripts of each interview were reviewed for quality then cross-checked against the Excel spreadsheet to confirm all data had been coded correctly. Changes to the diary were tracked in an item-tracking matrix [39, 40].

## Results

**Fig. 2:**
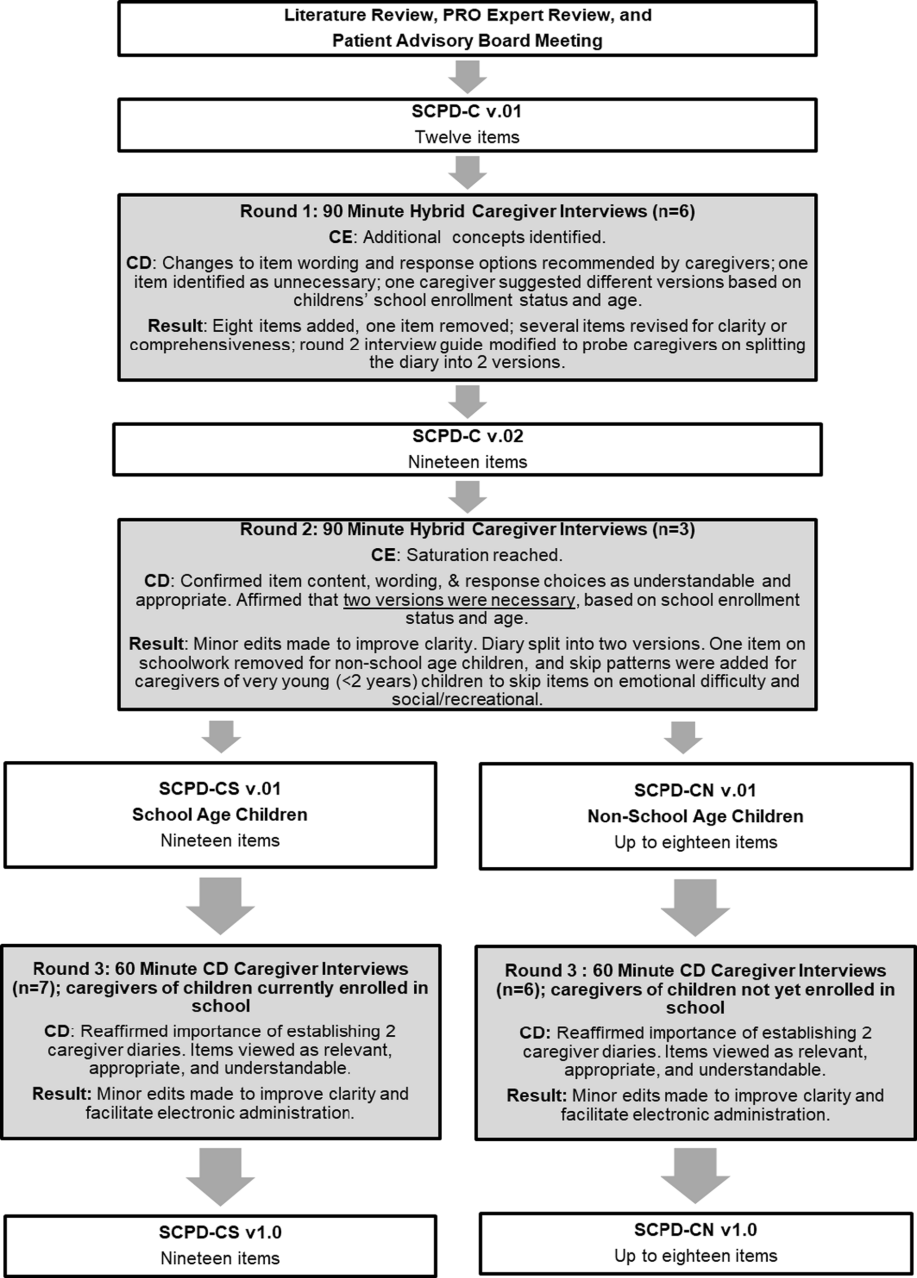
Revision history of the SCPD-CS and SCPD-CN. *SCPD-C* sickle cell pain diary-caregiver report, *CE* concept elicitation, *CD* cognitive debriefing, *SCPD-CS* sickle cell pain diary-caregiver report for school age children, *SCPD-CN* sickle cell pain diary-caregiver report for non-school age children

Results of all three rounds of caregiver interviews are presented below. In total, interviews were conducted with 22 caregivers (see Table 1 for demographic information). A summary of all revisions made to the diary as a result of feedback from the caregiver interviews is presented in Fig. 2.

**Table 1:** Characteristics of caregivers and their children with sickle cell disease

Demographic Information	Round One Hybrid Interviews n = 6	Round Two Hybrid Interviews n = 3	Round Three Cognitive Debriefing Interviews n = 13
Caregiver’s gender			
Male	1	1	2
Female	5	2	11
Caregiver’s education			
High school or equivalent	0	1	4
Some college	0	2	0
Associate’s degree	1	0	2
Bachelor’s degree	3	0	2
Post-graduate degree	2	0	5
Caregiver’s relationship to the child			
Parent	4	3	11
Grandparent	1	0	1
Cousin	1	0	0
Legal Guardian	0	0	1
Region of residence			
Northeast	3	0	3
Pacific	0	0	2
Southeast	3	0	6
Midwest	0	3	2
Child’s gender			
Male	2	2	4
Female	4	1	9
Child’s age			
Mean (SD)	8.3 years (2.8)	8.7 years (1.5)	5.1 years (3.1)
Range	3–11 years	7–10 years	11 months-11 years
Child’s disease type			
HbSS disease	2	0	8
HbSC disease	2	3	3
Don’t know/unsure	2	0	2
# of SCPCs in the past 12 months			
Mean (SD)	5.8 (4.2)	18.3 (2.9)	6 (5.9)
Range	1–12	15–20	1–24
Frequency of healthcare use for SCPCs			
Every time or almost every time	5	3	10
Sometimes but not all of the time	1	0	3
Treat at home/see regular doctor	0	0	0

SCD, sickle cell disease; SCPC, sickle cell pain crisis; SD, standard deviation

### Concept elicitation

During the CE portion of round one and round two interviews (n = 9), caregivers generally differentiated between every day experiences of SCD and experiences specific to SCPCs.

#### Symptoms

The two most frequently reported symptoms related to SCD observed on a day-to-day basis were pain and tiredness (both n = 5). SCD symptoms observed day-to-day included: fatigue, swelling, jaundice (all n = 2), constipation, bone aches, vomiting, headaches, loss of appetite, and shortness of breath (all n = 1).

All caregivers during round one and round two interviews (n = 9) reported observing their children experience intense pain during an SCPC, and that their children communicated their pain through verbal report or through nonverbal expressions and behaviors such as crying, moaning, screaming, irritability, or a change in posture. Other less common symptoms observed during an SCPC included lack of appetite (n = 3), vomiting (n = 2), dehydration, fatigue, jaundice, numbness, infection, and a high fever (all n = 1). SCPCs varied in length ranging from two hours to two weeks. The number of SCPCs observed by caregivers in a year also varied (range: 1–20).

#### Impacts and healthcare resource utilization

Caregivers reported numerous aspects of their child’s daily life that are negatively impacted by SCD (Table 2). Impacts were either the result of daily symptoms, or caregivers imposing limitations on their child due to concern of triggering an SCPC. Caregivers also reported impacts of SCPCs on multiple aspects of their child’s life (Table 3). Impacts were the result of SCPC symptoms (especially pain) or medical intervention.

**Table 2:** Daily impacts of sickle cell disease on children, as reported by their caregivers

Area of Impact	n (%) n = 9	Representative Caregiver Quote(s)
Emotional Health Anxiety Depression Anger Feeling judged Frustration	7 (78)	And it was, 'Why do I have to do this? Why do I have to go through this?' You know, 'Why me?' So, it affects her because now she's getting older and she's realizing what she's missing. She’ll see the… kids that don't have sickle cell get to run, and play, and jump, and do –so it affects her. She doesn't like sitting on the sidelines sometimes. So, you'll catch her crying. You’ll catch her upset
Social and Recreational Activities Modify or limit activities including: physical education, recess, field trips, or sports	6 (67)	He would like to play football, and that’s just not going to happen for many reasons. Because, just getting bruised, that can be an issue… I might take him shopping or do something kind of fun. But not too fun… any little thing can trigger him going into a crisis or just a lot of pain
Physical Functioning Limit physical activities including: running or playing	5 (56)	Sometimes she likes to run, and she likes to jump, and she likes to play; she loves to dance. So, with her, she gets winded so quick that she has to stop and sit down, and then she has to get back up and so… with sickle cell, you have a lack of oxygen you can pull into all of your organs, so with her, she’s off top like, OK, I can't go as long or I can't, but it doesn't stop her, but she’s going to try it
Daily Activities Interference in day-to-day activities General low activity	5 (56)	[He’s] not as active as my other kids. Um mainly you got to, “Come on let’s go,” you got to prep him up to get him to go to school and stuff like that
School Attendance School work Attentiveness during class	4 (44)	He’s literally falling asleep in class. He’ll come home and fall asleep and won’t finish his studies or his homework

**Table 3:** Impacts of sickle cell pain crises (SCPCs) on children, as reported by their caregivers

Representative Caregiver Quotes on SCPCs		
The pain crises. He would holler. Scream. Run through the house… Just more pain. The screaming, usually when he go through crises, everything starts to swell up. In his stomach, hands, lips, and ankles. And he don’t want to be bothered, so I know that’s when I really have to take him in When he’s in a crisis, it’s just intensifies about 10 times more. He’s screaming and in a lot of pain. And then sometimes—but the pain is kind of weird, because sometimes it’s like it’s certain body parts. It’s never in one area. Sometimes, 'My arm is so numb, and it hurts, and it feels like needles and pins sticking in.' And then there’s other times where he just, he has a really bad stomach ache, but he cannot hold anything down		

Impacts of SCPC	n (%) n = 9	Representative Caregiver Quote
Emotional Health Sadness Agitation/Irritability Anger Frustration Worry Fear	9 (100)	As bad as she want to [play with others], she’ll break down and cry… frustrated and angry. She gets so angry when you don’t let her, cause she be willing and wanting to
Sleep At home or at hospital Trouble falling asleep Trouble staying asleep	9 (100)	No one slept. Because when you're in the hospital, they're coming in and checking often, and it's just hard to get comfortable. Or we are waking him to give him the medicine…stay ahead of the pain, so then you have to try to get him to go back to sleep
School Attendance School work Attentiveness during class	8 (89)	She’s not able to take her test when she’s supposed to. She has to do make-up…and then sometimes she can’t catch—she’s not catching up because she has so much to do, and she’s a little bit behind
Social and Recreational Activities Unable to participate or needing to stop participation	6 (67)	Um, he likes to play basketball, and he’ll run, run, run, and then all of a sudden, he’ll stop, and I’m like, 'Why’d you stop? You know, finish it,' or whatever. He’s like, 'No… I think that’s enough. ‘I said… 'Why? ‘And he doesn’t really have a explanation for it. But I can look at him and tell it’s like, you know, 'I don’t want to, ' and I said, 'What is it? Are you afraid of being in pain or, you know, you think you might be in pain? ‘He’s like, 'Maybe.'
Physical Functioning Unable to complete physical tasks Immobile	4 (44)	I mean, he is not going to be climbing anything, that’s for sure, and he is not going to be jumping, because his bones are hurting. He is not going to be doing any of that, that’s why he just sits
Daily Activities Difficulty getting ready in the morning General inactivity Unable to use bathroom	3 (33)	No, he can’t move. No, I even have to help him to the bathroom. I even got him one of those urinals from the hospital. He has it in his room

Caregivers used different terms to describe SCPCs, including “pain crises” and “crises.”

Most caregivers indicated seeking treatment for their child’s SCPCs outside of the home every time or almost every time they experience an SCPC (n = 8). Six caregivers reported immediately seeking care in the emergency department after observing signs of an SCPC, such as change in behavior (e.g., screaming or crying), fever, or becoming immobile. All caregivers in rounds one and two (n = 9) reported medication use and other treatment during an SCPC, such as over-the-counter medications, prescription medications, intravenous therapy, and transfusions.

#### Caregiver burden during an SCPC

All caregivers described impacts on their own lives as a result of their child’s SCPCs (n = 9; Table 4). Work absenteeism (n = 7) was the most commonly reported impact. While some caregivers reported employers accommodated their need to miss work to care for their child, others described less flexibility in their work schedules. For these caregivers, missing work to care for their child meant the loss of income or risks to their overall employment status. Caregivers also described emotional health problems (n = 6) that resulted from watching their child experience SCPCs.

**Table 4:** Impacts and burden experienced by caregivers due to their children’s sickle cell disease

Caregiver burden	n (%) n = 9	Representative caregiver quote(s)
Work	7 (78)	I’m telling you, it’s hard. It’s really hard. My boyfriend is home with him now, and he plays a big role and everything. But if I have to leave work or I have to change something with my schedule, because he got ill at school or—anytime, it’s just anytime type thing
		I basically work my own schedule, but when I do get my schedule because we schedule a week ahead, I got to stick with that schedule. And if I don’t get them clients to—I end up paying [for] what day they miss, I don’t like it. If that fare costed $60, I have to pay that
Emotional health	6 (67)	Oh, so many times I have broke—I haven’t did it in front of her, but I have went in the bathroom, broke down because it’s like, this is my baby, and I can’t do nothing for to help her It’s very painful for the caregiver as well, it’s very painful for the caregiver, because sometimes when you know they are going through something, and you could see it on their face, and sometimes they just don’t want to be bothered, they are just sick of it themselves

#### Saturation

A saturation analysis was conducted to evaluate whether additional CE interviews were needed. Thematic saturation was reached by the 9th interview indicating no need for additional CE interviews. Specifically, in interviews 1–2, 36 concepts were identified; in interviews 3–4, 23 new concepts were identified. Ten new concepts were identified in interviews 5–6, 5 new concepts were identified in interviews 7–8, and no new concepts were identified in interview 9. The lack of new concepts in the final interview confirmed that 9 interviews were sufficient to reach saturation.

### Cognitive debriefing

#### Round one

Round one CD interviews (n = 6) tested the SCPD-C v.01. Overall, caregivers reported the diary was relevant, easy to answer, and would not be burdensome to complete during an SCPC, even if their child was in the hospital. All caregivers (n = 6) reported the initial instructions were clear, though a definition of the term “caregiver” was added. Instructions regarding daily experiences were added and edited for clarity.

Several items were revised in response to caregiver feedback (see Table 5 for a sample of SCPD-C v.01 items that were tested and modified as a result of round one interviews). Revisions were made to several items to increase ease of responding, reduce confusion, or provide a more complete set of response options. One item was removed from the diary entirely, while eight items were added (see Table 6 for examples of added items with quotes from caregivers). These revisions resulted in the SCPD-C v.02.

**Table 5:** Sample of items and response options modified in the SCPD-C v.01

Original Text (SCPD-C v.01)	Rationale for Modification	Caregiver Quote On Original Text	Final Modified Text (SCPD-CS v.01 and SCPD-CN v.01)	Caregiver Quotes On Final Modified Text
Item text: Did pain from your child's sickle cell pain crisis cause you to miss any work during the last 24 h? Response Options: **Yes** **No**	Caregivers suggested modifying response options to record how much of the work day was missed, and noted that sometimes they did not miss work because they were not scheduled to work that day, but otherwise would have missed work	If you wanted to streamline it, 'Did you miss a whole day? Did you miss a part of the day?' Because sometimes, if a sickle cell crisis starts, and your child is in school, and you have to go pick up your child, it might be halfway through the day	I missed all of my paid work I missed half or more (but not all) of my paid work I missed less than half of my paid work I did not miss any paid work I was not scheduled to go to work I do not currently work	I think they were clear. Because you can’t just have… three answers; you’re saying 'I missed all of my paid work' or 'I do not currently work' or 'I was not scheduled to work.' So the …answers… perfect
				I think those were fine. I think those made sense
Item text: During the last 24 h, how much, on average, did you observe signs of your child having a problem with pain not related to a pain crisis?	Caregivers found this item confusing and suggested that it be reworded for clarity	You don’t want to have to deal with worrying about reading something that’s long and drawn out or something that’s cumbersome, because it’s like why? You don’t want to deal with that. I just think it can be easier	Based on your observation, how much **on average** did your child display signs of **pain** during the past 24 h?	Yeah, I think it makes sense
				On a daily basis when he’s not in pain, that would make a whole lot of sense. It’s a relatively easy answer
Item text: During the last 24 h, did your child take any medication to control his or her pain?	Caregivers reported inconsistencies in how they interpreted “any medication”	I was thinking only medication I have at home and if the doctor gave me any. That’s medication period, it just says 'any medication'	Did your child take any medication to control his or her pain, including prescribed and over-the-counter medication, during the past 24 h?	It was very simple
				Yes, he did take medication, to control his pain. He took both over-the-counter medication, as well as, prescription pain medication
Item text: How long did the pain crisis episode last in total during the last 24 h? Select the time point that best represents the total duration (in hours and/or minutes)	Caregivers reported that it would be difficult to accurately provide the exact duration of time that a pain crisis lasts in hours and minutes	It can happen at any point in time. So, we could be anywhere, and I could look at her and be like, OK, something’s not right with her. I could be at church, I can be at school, I could be anywhere. Be in the mall, anywhere. And I can look at her and be like, 'What’s wrong with you?' And then, most of the time by the time I can do that she's already been in pain probably a good hour or two and just hasn’t said anything	**Approximately** how many hours did the pain crisis last during the past 24 h?	No, not too hard. It was just more so, just, yeah, it was just more so me just trying to figure out roughly about what time she woke up and what time she went to bed so I was just trying to get those answers, you know, get the number of hours
				That was easy
				Yeah. I can estimate

**Table 6:** Sample of items added to the SCPD-C v.01 as a result of caregiver feedback from Round One

Rationale	Representative Caregiver Quote	Item Added to the SCPD-C v.02
All 6 caregivers reported that SCPC**s** had an impact on their child’s emotional health	I would ask, ‘How does sickle cell affect the persons as a whole, emotionally? What are the thoughts behind a person with sickle cell, they are feeling?’ Things like that, because when you go into those—and you ask someone, I am sure if they were asked, because I ask them all the time, and he just wish it didn’t happen. Most time they wish it didn’t happen, and the main question that I get is, ‘Am I going to die?’	Based on your observation, how much emotional difficulty did your child experience due to his or her sickle cell disease during the past 24 h (for example, being irritated or mad, worried or afraid, or feeling very sad)?
Four of 6 caregivers reported an impact on their emotional health as a result of their child’s SCPC	I think sometimes it affects the parent, so I know you've given the gauge in how it affects the child… it affects the parent watching the child go through it. So that’s another part… I’m so sorry. I’m sniffling	How much emotional difficulty did you experience due to your child’s sickle cell pain crisis during the past 24 h (for example, stress, anxiety, sadness, or depression)?
Caregivers discussed a tendency to go to the hospital when their child is experiencing a pain crisis. Two of 6 caregivers endorsed adding an item related to healthcare utilization	I can't count how many times we have gone to the emergency department. In the last year usually, it's just a blood test, they say it's a common cold or some little virus and they send us home, but for a while it meant an overnight stay for a couple days	Did the pain crisis lead to a visit with your child's primary care doctor, urgent care center, or emergency department during the past 24 h?

One caregiver reported that items regarding school attendance, school work, and daily activities (including chores, social and recreational activities) were not appropriate for her to complete because her child was three years old and did not yet attend school or do the same types of activities as older children. She suggested removing these items when the diary is to be completed by a caregiver of a child not yet in elementary school. This feedback suggested that two different diaries would be most appropriate: one for caregivers of young children not yet attending school, and one for caregivers of school-age children. To explore this insight, the interview guide for round two was modified to specifically ask caregivers whether splitting the diary into two versions would be necessary.

#### Round two

The purpose of round two interviews (n = 3) was to evaluate the SCPD-C v.02, confirming that no additional concepts needed to be included in the diary, and evaluating the clarity and appropriateness of all items and response choices.

Overall, caregivers confirmed the relevance and comprehensiveness of the diary items and initial instructions. Minor edits were made to the wording of various items to improve clarity. One item’s response options were edited based on caregiver feedback.

Caregivers in round two affirmed the diary should take into account the age of the child and whether or not they are attending school. Based on caregiver feedback, the diary was split into two versions: the SCPD-CS v.01 for school-age children and the SCPD-CN v.01 for non-school age children. An item was added to the start of the diary asking if the child currently attends school; this item ensures administration of the correct version during electronic implementation.

The SCPD-CN v.01 mirrored the SCPD-CS v.01 with two primary exceptions. One item on interference with schoolwork was removed. Additionally, an item was added asking the age of the child, and skip patterns were added in the SCPD-CN v.01 to allow caregivers of children under the age of two to skip items that are difficult to answer based on the child’s age. Specifically, caregivers of children < 2 years old would now skip items related to their child’s emotional difficulty and interference in activities of daily living and social/recreational activities. These changes allow the SCPD-CN to be appropriate for all non-school age children, regardless of age.

#### Round three

Round three interviews used only cognitive debriefing techniques and evaluated the two versions of the caregiver diary separately. Seven caregivers of children < 12 years of age who were currently enrolled in school completed and evaluated the SCPD-CS v.01. Six caregivers of non-school-age children evaluated the SCPD-CN v.01. These interviews reaffirmed the importance of establishing two caregiver diaries to capture the unique experiences of children with SCD enrolled in school as compared to children with SCD not yet enrolled in school. Items were viewed as relevant, appropriate, and understandable to caregivers of children < 12 years old. No new items were added. Minor edits were made to the diaries to increase clarity, accuracy, and comprehension. For example, an edit was made to clarify the definition of “school,” in order to specify that school includes kindergarten and all subsequent grades. Additional minor changes were made to the diaries to facilitate electronic administration, resulting in the SCPD-CS v1.0 and the SCPD-CN v1.0.

## Summary of final diary content

As a result of the three rounds of caregiver interviews, 19 items were included in the SCPD-CS v1.0 and 18 items were included in the SCPD-CN v1.0. The final diaries measure pain severity, pain duration, fatigue, and medication use and HCRU in children with SCD to manage pain, and impact of pain on the child’s HRQoL during an SCPC, as observed by the caregiver (Table 7). The final diaries also include non-SCPC related pain and its impact on the child’s HRQoL observed by the caregiver during times when signs of an SCPC were not observed in the past 24 h.

**Table 7:** Final content: Sickle Cell Pain Diaries (SCPD-CS v1.0 and SCPD-CN v1.0)

Item Content	SCPD-CS v1.0 Caregiver Report, School Age	SCPD-CN v1.0 Caregiver Report, Non-school Age
**For Electronic Administration**		
Age of child	1 item	1 item
School enrollment	1 item	1 item
**During an SCPC: Presence, Duration, and Severity**		
Observed signs of SCPC	1 item	1 item
Duration of SCPC	2 items	2 items
Healthcare utilization due to SCPC	1 item	1 item
Medication use due to SCPC	2 items	2 items
**Observed Interference of SCPC-related Pain on Daily Activities**		
*Interference with…*		
Child’s school attendance	1 item	1 modified item: Preschool or Daycare
Caregiver’s paid work attendance	1 item	1 item
Child’s schoolwork	1 item	–
Child’s activities of daily living	1 item	1 item: Only if the child is 2 years or older
Child’s social and recreational activities	1 item	1 item: Only if the child is 2 years or older
Child’s sleep	1 item	1 item
**Observed Impacts of SCPC-related Pain on Fatigue and Emotional Difficulty**		
Fatigue	1 item	1 item
Child’s emotional difficulty	1 item	1 item: Only if the child is 2 years or older
Caregiver’s emotional difficulty	1 item	1 item
**Outside of an SCPC: Observed Pain, Fatigue, and Impacts of SCD**		
Signs of pain outside of an SCPC	1 item	1 item
Interference with sleep	1 item	1 item
Fatigue	1 item	1 item
Emotional difficulty	1 item	1 item: Only if the child is 2 years or older

## Discussion

The goal of this study was to evaluate the content validity of an ObsRO measure diary (SCPD-C) for caregivers of children with SCD aged < 12 years. Early caregiver interviews revealed that the impacts and experiences of school-aged children differed meaningfully from those of non-school-aged children. As a result, the diary was split into two versions: the SCPD-CS (for school-aged children) and SCPD-CN (for non-school-aged children). Both diaries include questions related to the pain, sleep, and fatigue on days the child is not experiencing an SCPC; additional questions related to SCPC duration, treatment, impacts, and interference with various daily activities are administered on days the child is experiencing an SCPC. Both versions of the diary also provide the opportunity for the caregiver to report on impacts they have experienced as a result of their child’s SCD. The precise item content is tailored based on the child’s age and school enrollment status. Both diaries capture the daily impact of SCD and have the ability to capture the variability within and across SCPCs. The inclusion of items related to HRQoL allows for a broader characterization of a patient’s health status, moving beyond what can be assessed by HCRU data alone, ultimately providing a more holistic view of the impact of SCPCs and SCD on children.

Caregivers described the burdensome impact SCD and SCPCs have on their child’s overall HRQoL. Concepts that emerged as especially important were the impact of SCD on the child’s emotional health, social and recreational activities, physical functioning, daily activities, and school (both school attendance and schoolwork). These same issues were further compounded by the impact of SCPCs; and SCPCs additionally impacted sleep. In addition to the myriad of impacts on HRQoL experienced by children with SCD, caregivers reported a high rate of HCRU for SCPCs as compared to published HCRU rates of children with SCD [7, 41, 42]. Given that healthcare visits generally increase with age in children with SCD up to early adulthood [43], these findings support the importance of capturing children’s healthcare experience and impacts on HRQoL. Caregivers themselves experienced substantial emotional and work-related burdens as a result of caring for their children during an SCPC.

These findings should be interpreted in the light of some limitations. One limitation is that this study tested a paper version in all three rounds of interviews; future work should include usability testing of the programmed survey in electronic format. An electronic administration, such as on a smartphone or tablet, will allow for management of skip patterns, reminders to complete the diary, and include other features that will make it easier for caregivers to complete the diary.

Additionally, the inclusion of various types of caregivers (e.g., legal guardian, single parent, independent caregiver) and additional male caregivers is recommended in future work as our sample included 18 parents and 18 female caregivers. Seventeen caregivers in our sample had at least some college education. Future studies should include caregivers with a greater mix of education levels. Additional validation testing in ex-US samples should be conducted if the diaries will be used outside the US.

Strengths of this study include having a rigorous research design with many in-person interviews in multiple regions, a large sample size given SCD is a rare condition, and input from expert clinicians, and a patient advisory board in the instrument design phase. The sample size of this study was guided in part by saturation analysis findings, and by literature recommendations for instrument development. Thematic saturation was met after the 9th interview. Additional cognitive debriefing interviews (round three) were needed to test that the SCPD-CS and SCPD-CN were appropriate, easy to understand, and relevant to caregiver observed experiences.

While some measures, such as the PedsQL Sickle Cell Disease Module, have been developed and tested to capture the impact of SCD symptoms on children’s with SCD HRQoL [44, 45], this is the first daily diary with evidence of content validity that is designed for a caregiver to report for children with SCD ages < 12 years. When children have SCPCs, they are often unable to move or think clearly, and completing a survey themselves is not feasible. Caregivers are able to observe the impact of SCD and SCPCs on children and report daily. The content of the diaries is similar to the PedsQL Sickle Cell Disease Module as the diaries also capture the pain impact, emotional impact, and treatment management of pain. The ability to report daily is important given the fluctuating nature of SCPCs in terms of duration, severity, and impacts within and across individuals. Additionally, the diaries’ capture caregiver’s burden during their child’s SCPC event. Clinicians (from the day-long patient advisory board meeting) and caregivers felt having an available daily diary, preferably completed on a smartphone or other electronic device, would be useful to improve communication with clinicians and hospitals and improve patient care.

While these findings support the content validity of the SCPD-CS v1.0 and SCPD-CN v1.0, additional work including development of scoring algorithms and user’s manuals, and conducting psychometric evaluation of the diaries with larger sample sizes will be needed. Use of the diaries is anticipated in future clinical trials with caregivers of children who experience SCPCs as a way to capture the HRQoL impacts of new interventions designed to reduce the frequency and intensity of SCPCs.

## Conclusions

The SCPD-CS v1.0 and SCPD-CN v1.0 are appropriate and fit-for-purpose ObsRO measures of SCPC-related pain frequency and severity and the impacts on HRQoL. This study highlights the important of including caregiver input when developing a pediatric ObsRO.

## Data Availability

Specific data points can be made available upon reasonable request, due to the complex qualitative nature of the study design.

## Abbreviations

SCD: Sickle cell disease
SCPC: Sickle cell pain crisis
SCPD-C: Sickle Cell Pain Diary-Caregiver Report
SCPD-CS: Sickle Cell Pain Diary-Caregiver Report for School Age Children
SCPD-CN: Sickle Cell Pain Diary-Caregiver Report for Non-School Age Children
US: United States
HRQoL: Health-related quality of life
HCRU: Healthcare resource utilization
ObsRO: Observer-reported outcome
CE: Concept elicitation
CD: Cognitive debriefing
